# The Study of Medicine

**Published:** 1903-09-05

**Authors:** 


### THE STUDY OF MEDICINE.

The significance and possibilities of a life spent in the study and practice of medicine have, during recent months, been subjects of anxious debate in not a few households, and now, for good or ill, the decision has been taken and the new recruits are about to take service under the banners of their choice. The influences and considerations which have determined the issue have doubtless been of a very varied character, and in most instances, probably, the resolution rests on somewhat mixed motives. That in every case the future will justify all the anticipations that have been formed would be too much to expect. The high and hopeful outlook of youth must in the nature of things suffer some eclipse. But there are great possibilities and opportunities for those of cheerful courage and resolute endeavour, and the new generation may be sure that the gentle wishes of their seniors go with them to their trial.

In its external aspects, or relation to the community, medicine is an art concerned with the relief of suffering and with the prevention and cure of disease. Both the State and the individual citizen find the need of its guidance and aid. It touches closely large measures of policy which concern the general welfare, and it is equally in contact with some of the most sacred interests of private life. Both as a public adviser and servant, and as a personal counsellor and friend, the medical practitioner holds a special and distinctive position. To exercise these considerable responsibilities is a task well worthy of the best abilities which culture can command, and the opportunity of so extensive and varied a platform may be claimed as a peculiar attraction of the profession of medicine. The strong and steady brain whose delight it is to bear burdens and to feel the glow of the strenuous life may be sure that the stimulus of a sustained purpose and of large occasions will not be wanting. To meet all this it may at once be said that medical work efficiently performed brings some measure of public recognition and reward. And, to present it in its narrowest outlook, it is certainly a creditable and worthy method of gaining a livelihood. These several external aspects of medicine have their legitimate influence in attracting workers into its ranks. It is, however, our more immediate concern to regard the study of medicine from a personal or, rather, a subjective standpoint, and to consider the influences it is likely to exert on those who pursue it, and the opportunities it affords for the development and realisation of individual character and ability. It is a just consideration to ask whether or not medicine is a profitable field for the cultivation of good mental and moral qualities. From this point of view it may be stated that the student of medicine has the chance to enjoy the full benefits of a scientific atmosphere. His work commences with the physical and biological sciences, and it will be his own fault if the spirit and methods of his earlier experiences are not made to control all his later opportunities. Now the distinguishing marks of a scientific training may be said to be accuracy in observation and statement, and thoroughness in method and work. The development of these qualities is the first step in medical education. The necessary discipline often appears to the student a barrier to the entry on actual medical and surgical work, whereas, in truth, it is the very means by which he is prepared to undertake such work on sound principles and with advantage to himself. His later life is largely to be spent in the observation and interpretation of facts and in the endeavour to appraise the value of statements in which a non-scientific public describes its disabilities and sufferings. It is the discipline of science which gives the ability to recognise the value of evidence, and to estimate exactly how it affects particular propositions. That is a valuable end to gain. To distinguish between names, opinions, and fancies, on the one hand, and facts, events, and evidences, on the other, is to be able to preserve a free mental outlook, and to be saved from the darkening influence of words without knowledge. But a scientific discipline is not limited to the observation and classification of facts or to the definition of principles. It introduces the student to the larger questions of philosophy. In the present day the scientific worker is not content merely to provide materials and devise processes having a utilitarian value. Nor can he leave the speculative questions arising out of his discoveries to the discussion of those who deem philosophy their peculiar province. On the contrary, his methods and principles have invaded this province, and his experiences compel the necessity of considering their interpretation and significance. His outlook is not limited to the laboratory and workshop. It commands the entire field of human thought. To say, as one sometimes hears, that the study of medicine cramps the imagination and develops a narrow view of life, argues a very imperfect acquaintance with the discipline and opportunities in which such study abounds.

In its cultivation of the ability to observe and to state facts with accuracy, medicine has a common ground with other forms of scientific training. But it may claim a special position in that it associates the influence of science with the demands of practical life and with the living interest of human personality. It is not with mere formulas that the student of medicine is engaged, and he is no more shut up to the rigid facts of the laboratory than he is permitted to dream in the seclusion of the study. Chemistry and physics and the allied sciences give him, it is true, his sure footing, but he must carry his knowledge of these so as to permit him to apply it among the idiosyncracies, the frailties, and the emotions of individual men and women. His work, in short, is touched at all points with human interests and sympathies. The necessity for shrewd judgment, firm decision, and resolute action, is pressed upon him, and with all these there come the gentle and generous influences which make the whole world kin. Opportunity for kindliness is on every hand, and also for sane and manly sentiment. It is not to be wondered at that a career so rich in opportunities for developing scientific precision, philosophic breadth, and true manliness should attract to its ranks ardent and generous youth. The study of medicine is not merely the work of a few years; it endures for a lifetime. But habits grow from the outset, and those who would greatly attain must make their beginnings sure.

